# From Synapse to Function: A Perspective on the Role of Neuroproteomics in Elucidating Mechanisms of Drug Addiction

**DOI:** 10.3390/proteomes6040050

**Published:** 2018-12-09

**Authors:** Luis A. Natividad, Matthew W. Buczynski, Daniel B. McClatchy, John R. Yates

**Affiliations:** 1 Department of Neuroscience, The Scripps Research Institute, La Jolla, CA 92037, USA; 2 School of Neuroscience, Virginia Polytechnic Institute and State University, Blacksburg, VA 24061, USA; mwb@vt.edu; 3 Department of Molecular Medicine, The Scripps Research Institute, La Jolla, CA 92037, USA; dmcclat@scripps.edu

**Keywords:** neuroproteome, drug abuse, neuropeptidomics, phosphorylation, interactome

## Abstract

Drug addiction is a complex disorder driven by dysregulation in molecular signaling across several different brain regions. Limited therapeutic options currently exist for treating drug addiction and related psychiatric disorders in clinical populations, largely due to our incomplete understanding of the molecular pathways that influence addiction pathology. Recent work provides strong evidence that addiction-related behaviors emerge from the convergence of many subtle changes in molecular signaling networks that include neuropeptides (neuropeptidome), protein-protein interactions (interactome) and post-translational modifications such as protein phosphorylation (phosphoproteome). Advancements in mass spectrometry methodology are well positioned to identify these novel molecular underpinnings of addiction and further translate these findings into druggable targets for therapeutic development. In this review, we provide a general perspective of the utility of novel mass spectrometry-based approaches for addressing critical questions in addiction neuroscience, highlighting recent innovative studies that exemplify how functional assessments of the neuroproteome can provide insight into the mechanisms of drug addiction.

## 1. Drug Addiction: A Dysregulation of Plasticity in Motivational Circuitry

Drug addiction is a chronic relapsing disorder characterized by compulsive drug-seeking, the loss of control in limiting drug intake and the emergence of negative emotional states during drug abstinence [1]. In the past few decades, substantial research has contributed to our understanding of the underlying circuitry that influences addictive behaviors. The transition from casual use to dependence is mediated by changes in multiple interconnected brain systems involved in the processing of reward, stress, hormonal regulation and cognitive function [2]. Collectively, these molecular changes drive synaptic plasticity and alter connectivity between these brain regions, thus influencing a multitude of maladaptive behaviors that have come to characterize substance use disorders.

Drugs of abuse are widely recognized to facilitate dopamine transmission in brain reward systems, contributing to a powerful hedonic and euphoric response that reinforces drug-taking behavior. As drug use continues, increased dosage and frequency of use induces pivotal changes in brain reward pathways (e.g., the mesolimbic dopamine system) such that the same circuits recruited initially respond differently upon re-exposure. Pharmacological manipulation of the dopamine system has long been recognized to reduce drug intake in preclinical models of addiction [3,4]; however, there are multiple reasons why blocking this system is problematic in the clinical setting [5]. More generally, due to the disruptive side effects in mood (dysphoria) and motor-based (tardive dyskinesia) function of dopamine receptor antagonists, the lack of patient compliance remains a substantial problem. Conversely, dopamine receptor agonists reduce drug intake but also facilitate signs of drug-seeking in preclinical models, underscoring the potential for provoking drug relapse. The identification of non-dopaminergic targets may therefore provide an alternative for therapeutic treatment of addictive disorders.

Although the deregulation of brain reward remains a critical symptom of addiction, the maladaptive behaviors exhibited by addicts during drug abstinence are indicative of a more complex pathology driven by changes in additional brain structures. While a diverse set of hypotheses have been developed to explain the transition from casual use to dependence [2,5,6,7,8], “neuroplasticity” often emerges as a common theme linking these ideas. From this perspective, addictive behaviors emerge from a collection of neuroadaptations in specific neuronal circuits and neuroanatomical regions. By understanding the molecular changes that influence drug-induced neuroplasticity, it may be possible to stall or reverse these changes through a combination of cognitive behavioral therapy and small molecule treatments. For these reasons, developing a more comprehensive understanding of the molecular changes that occur in the brain following chronic drug use remains an important step in treating addiction.

## 2. Identification of Druggable Targets for Treating Addiction Using a “Neuroproteomics” Approach

While the population of drug-addicted individuals continues to grow in the United States and worldwide, the small number of available treatment options has failed to address this growing burden. Drugs that are FDA-approved for other diseases have been evaluated off-label to treat addiction, yet have been met with limited success, highlighting a critical need to identify addiction-specific changes in the central nervous system (CNS) that can be harnessed for clinical therapeutics.

**Figure 1:**
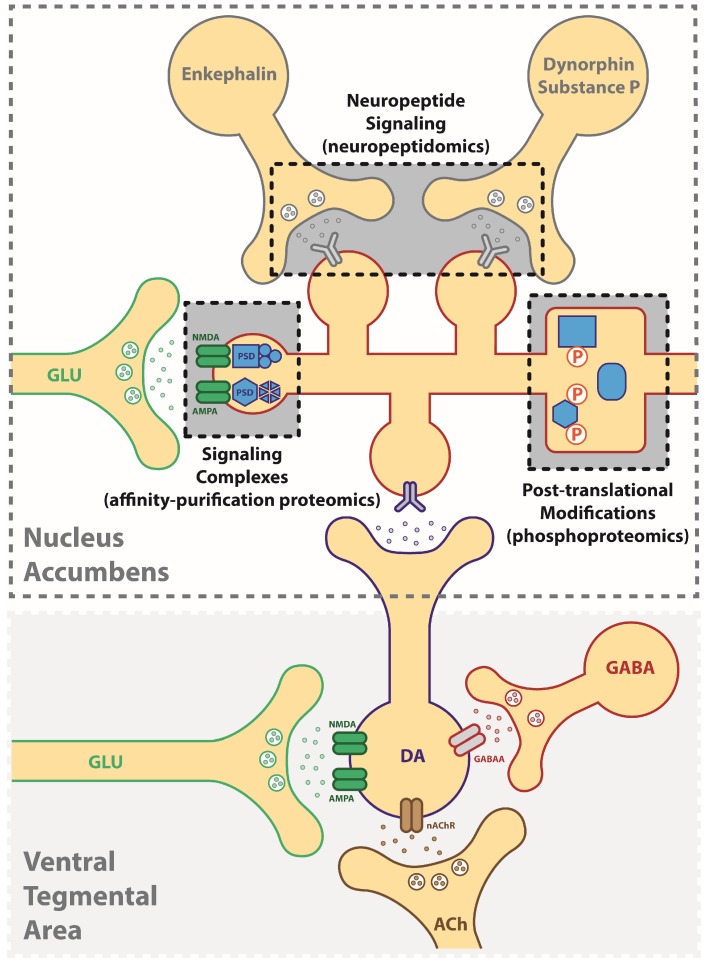
Application of neuroproteomic assessments in the study of addictive disorders. Synaptic plasticity underlying addiction-related behaviors can result from changes in (1) neuropeptide signaling (neuropeptidomics), (2) signaling protein complexes (affinity-based proteomics), (3) post-translational modifications such as phosphorylation (phosphoproteomics). DA: dopamine, GLU: glutamate, ACh: acetylcholine, GABA: gamma-aminobutyric acid.

From a pre-clinical perspective, a better understanding of the molecular alterations in the CNS would offer a wealth of information regarding the mechanistic underpinnings of disease, products of disease pathology (biomarkers) and the identification of high-value targets for the development of precision medicine. In the simplest case, a small molecule therapeutic would selectively bind a protein target in order to intercept a molecular change that underlies an important behavioral construct, such as drug reinforcement. Here, we depict the brain reward pathway (Figure 1) consisting of dopamine neurons in the ventral regions of the midbrain that project onto medium spiny neurons in the nucleus accumbens, where an increase in dopamine release is associated with the positive hedonic qualities of abused drugs. A broad-scale investigation of these regions would be useful not only for identifying changes in protein expression (e.g., proteomics) but also for capturing aspects of the proteome that confer functional changes. This review will discuss the following neuroproteomic strategies that are currently being employed to elucidate this dynamic signaling network: (1) changes in protein-protein interactions (affinity-purification proteomics), (2) post-translational modifications (e.g., phosphoproteomics) that alter cellular signaling pathways and (3) *in vivo* monitoring of signaling peptides (neuropeptidomics). In each of these cases, proteomics offers a valuable means to identify targets in a more unbiased manner than conventional protein assays, and together they establish a foundation for the “hit-to-lead” optimization of novel druggable targets for addiction and related psychiatric disorders. Here, we demonstrate the value of applying a proteomics approach to the CNS by highlighting recent studies that utilize novel methods for elucidating the mechanisms of addictive disorders.

## 3. Proteomics: Identifying Druggable Targets from Changes in Protein Expression

Traditional proteomic approaches have evaluated broad-scale changes in protein abundance in the brain following chronic drug exposure. The results have yielded a plethora of information on candidate targets, summarized elegantly in review articles for alcohol [9], morphine [10], and other psychostimulants [11]. Bottom-up proteomic strategies have greatly expanded the ability to identify the proteins in complex sample mixtures via the enzymatic digestion of proteins to generate peptides which are fragmented in the mass spectrometer [12]. Search algorithms (e.g., SEQUEST, Mascot) then match the fragmentation patterns of the peptides against theoretical spectra generated from protein databases, controlling for false positives with decoy strategies [13]. Thus, to maximize the number of peptides analyzed in biological mixtures, several components are often emphasized in method development: (a) sample protein preparation, (b) peptide fractionation (c) mass spectrometer acquisition, and (d) bioinformatic processing of the generated spectra. These approaches have been described at length in many excellent neuroproteomic reviews [14,15,16,17,18], and below we will mention a few issues regarding their implementation in addiction studies.

### 3.1. Current Limitations of Proteomics in Addiction Research

Obtaining a viable sample that is likely to contain relevant targets of addiction poses a critical challenge for neuroscientists. While anatomical structures provide some level of specificity, there is substantial heterogeneity at the molecular and behavioral level. An interesting example of this involves the dorsal striatum known to contain afferent dopaminergic terminals that are activated by drugs of abuse. While this structure is often dissected and analyzed as a singular region, site-specific inactivation has informed the rationale for distinguishing critical areas. In this regard, the dorsal medial region is important during acute drug exposure given its role in influencing goal-directed behavior that establishes early drug-cue associations; however, the dorsal lateral region becomes increasingly important as addictive behaviors become more automated, thus reflecting one of the core symptoms of addiction that influence compulsive intake [19,20,21]. Relatedly, methamphetamine only activates about 5–10% of neurons, suggesting that there may be a diluting component by the inactive majority when assessing whole tissue [22]. Moreover, subcellular locations within the brain are more likely to contain the sites of action of drugs of abuse (e.g., membrane/synaptic proteins, synaptosomes), suggesting that the enrichment of these compartments may result in a more viable fraction for further study. These technical aspects, as well as novel methods for evaluating the synaptosome [23], have been reviewed in much detail, yet the practical consideration remains that fractioning the proteome reduces the amount of starting material available. Thus, implementing a proteomics assessment often requires a delicate balance between the amount of protein required to identify a significant change and the amount that may be feasibly collected from an experimental preparation. For these reasons, the number of proteomics studies evaluating the effects of drug dependence remains relatively small when compared with other biological fields such as cancer and inflammation.

### 3.2. Identification of Important Changes in Expression

Identifying a druggable target from the many changes observed in the proteome remains a persistent challenge in neuroproteomics research. One strategy is to employ pathway analyses that can distill large amounts of information into known signaling networks, biological functions and associated disease states. A notable example from Salling et al. identified 29 proteins that were dysregulated by moderate alcohol drinking in the mouse amygdala using two-dimensional difference gel electrophoresis (2-DIGE) [24]. Ingenuity Pathway Analyses (IPA) revealed that many of these proteins annotated to neuronal signaling (CNS cell signaling, 14 proteins) and morphology (cell morphology, 8 proteins), while others were annotated to synaptic (excitatory plasticity, 7 proteins) and neurobehavioral (psychiatric disorders, 6 proteins) changes that were dysregulated by alcohol exposure. Of the total proteins identified, only calcium/calmodulin-dependent protein kinase II (CaMKII) was detected in each of these clusters, providing powerful support for the suggestion that this kinase plays a critical role during the early stages of alcohol dependence. In a separate study, Reissner et al. [25] used a BisoGenet plugin in Cytoscape to elucidate a glutamatergic signaling network based on the quantification of 42 proteins obtained from the nucleus accumbens of cocaine self-administering rats. Notably, the A-kinase anchoring protein 5 (AKAP5) located in the postsynaptic density was upregulated along with parallel changes in membrane-associated guanylate kinase markers such as discs large homolog-associated protein 3 (PSD-95). Follow-up studies applied the use of interfering peptide constructs to dissociate the interaction between AKAP and binding sequences for protein kinases that normally promote the insertion of ionotropic glutamate receptors, such as α-amino-3-hydroxy-5-methyl-4-isoxazolepropionic acid (AMPA).

The emergence of sample-labeling and multiplexing procedures provides an added dimension of research analysis of the brain proteome. Several strategies currently exist and are well documented in the literature (e.g., fluorophore, isobaric and metabolic labeling), each displaying their own unique advantage in terms of reducing the technical variation between mass spectrometric analysis and other aspects of labeling efficiency and sample preparation [26,27]. The recent emergence of multichannel labeling kits (e.g., iTRAQ and TMT) offer an attractive feature for neuroscientists who commonly employ more than two experimental groups. From an etiopathological perspective, it is feasible to gain insight into conditions that may result in discrepant changes at different stages of disease progression. For example, Lull et al. [28] utilized fluorophore labeling to compare the prefrontal cortical proteome in cocaine self-administering rats with 2-DIGE methods. The analysis comparing naïve versus cocaine-exposed rats experiencing acute or chronic abstinence yielded a total of 20 significant changes (e.g., synaptosomal-associated protein 25, dynamin-1), revealing a temporal pattern of protein expression classified as either (a) drug-induced changes that persisted into abstinence, (b) drug-induced changes that did not persist or (c) unique changes attributed exclusively to the abstinence period. The distributed pattern of expression argues against comprehensive treatment strategies that may miss a critical therapeutic window in which addicted individuals may be more or less prone to displaying altered cortical mechanisms. Nimitvilai et al. also investigated changes in the synaptosome in the orbitofrontal cortex of heavy alcohol-drinking macaque monkeys, a cortical region strongly implicated in decision-making and relapse [29]. iTRAQ labeling procedures identified 57 distinct protein changes in the synapse resulting from chronic alcohol exposure. Similar to Salling et al. [24], IPA analysis of all the differentially expressed proteins indicated a strong impact of alcohol on networks involved in cell-to-cell signaling, including a number of proteins that overlapped with glutamatergic function. Collectively, these studies demonstrate that synaptic transmission and excitatory signaling complexes represent important targets for understanding and treating dependence and addiction.

### 3.3. Confirmation of Targets Identified by Proteomics

A common strategy in proteomics research is to seek confirmatory evidence/validation of the targets derived from large-scale analyses with antibody-based molecular approaches. For instance, Nimitvilai et al. confirmed changes in a subtype of glutamate AMPA receptors (GluA1) in alcohol-dependent macaques using traditional western blot techniques [29], as validated antibodies exist for this target. However, confounds associated with commercial antibodies for western blot analysis, including issues with reliability (batch-dependent variations) and selectivity (cross-reactivity and antigen/epitope binding) [30], often render this technique as somewhat restricted to previously implicated targets with a limited capacity for identifying novel targets. To validate potential hits, many groups also rely on corresponding alterations in transcript levels as a correlate of central dogmatic principles. However, transcriptional changes often fail to reflect protein levels in CNS tissue and thus offer less predictable face validity than direct protein measurements. For this reason, targeted mass spectrometry approaches such as selected reaction monitoring (SRM) analysis have gained popularity for the confirmation and quantification of specific changes in the brain proteome [15,31].

To realize the ultimate goal of identifying druggable targets, the proteomics field would benefit from employing multiple strategies in elucidating proteome-derived targets. Exploring functional relevance with respect to whole organism behavior would enhance the translational value and application to the clinical population in question. For example, Salling et al. expanded on the initial proteome work in alcohol-drinking mice to elucidate a functional role for increased CaMKII subunit α expression in the amygdala, providing molecular and electrophysiological evidence of the strengthening of long-term potentiation (LTP) signaling ostensibly driven by CaMKII binding sites on GluA1 receptors [24]. These findings served as an impetus for site-anatomical procedures in which CaMKII inhibitors were injected directly into the amygdala and shown to have an ameliorating effect on symptoms of alcohol reinforcement, escalated drinking and relapse sensitivity [32,33,34]. Reissner et al. and other work from the Kalivas group have also applied the use of small-peptide inhibitors [25] and antisense oligonucleotides [35] to elegantly demonstrate both biological and behavioral roles in the tempering of cocaine reinstatement and drug-seeking behavior. Employing behavioral approaches may also distinguish between unforeseen predictions of applied treatment. For example, Chen et al. identified a network of proteins in the hippocampus that annotated to cyclin-dependent kinase 5 (CDK5) and ras homolog family member B (RhoB) signaling, both of which were upregulated in heroin self-administering rats [36]. Interestingly, the local infusion of a CDK5 inhibitor enhanced heroin intake, whereas a RhoB inhibitor reduced indices of heroin-seeking behavior as opposed to direct effects on self-administration. The authors concluded that CDK5 upregulation may have been a compensatory effect of repeated heroin exposure, whereas RhoB is likely to contribute to the sensitization of environmental cues that influence relapse. Collectively, this body of work highlights the inherent value of implementing multiple strategies for elucidating downstream pathways and target mechanisms of addiction.

## 4. Affinity-Based Proteomics: Protein Interactome as an Approach for Targeting Mechanisms of Addiction

Recent advancements in the isolation of neuronal circuits using chemogenetic or optogenetic tools clearly demonstrate the heterogeneity of brain structures involved in addiction [37,38]. Even within classifications of neuroanatomical structures, multiple overlaid circuits modulate distinct, and often opposing, behavioral responses. Synaptic activity-induced changes in receptor signaling complexes can drive neuroplasticity, which underlies behavioral abnormalities. Importantly, the discrete disruption of signaling complexes may circumvent the inherent side-effects often produced by pharmacotherapies that globally affect excitatory or inhibitory neural processing. For this reason, identifying relevant changes in protein signaling complexes represents an important approach in the optimization of potential druggable targets.

While substantial work has demonstrated an important role for nicotinic receptors in the development of nicotine dependence by targeting the ligand binding site, the proteins they interact with (i.e., the interactome) also participate in many important synaptic and immunological processes. Thus, drug-selective signaling complexes may represent unique opportunities for pharmacological intervention to disrupt addiction-related behaviors while minimizing therapeutic side-effects inherent in directly targeting nicotinic receptors. One such receptor complex in the hippocampus facilitates nicotine relapse-like behavior in rats [39]. In this study, Lui et al. demonstrated the formation of a complex between nicotinic α7 receptors and the glutamate receptor *N*-methyl-d-aspartate (NMDA) that is abundantly expressed in the hippocampus. To functionally evaluate this complex, they developed and validated small peptides that interfered with the formation of this complex without altering receptor-mediated signaling by either nicotinic α7 and NMDA. The intracerebroventricular delivery of this interfering peptide to the brain prevented the reinstatement of nicotine self-administration in rodents, providing evidence that this complex represents a viable druggable target for nicotine addiction.

Affinity-based enrichment can also provide insight into the network of protein interactors that may alter cellular signaling. For example, Wills et al. immunoprecipitated the NMDA receptor NR2B in hippocampal preparations to reveal a network of interacting proteins including scaffolding and PDZ-domain binding proteins that were dysregulated by chronic alcohol exposure [40]. Of the 64 proteins identified in synaptic fractions, the long-term depression (LTD) markers Arc and Homer 1 were observed to be upregulated, while the AMPA receptor GluA2 was downregulated in alcohol-dependent mice. The findings provided a basis for follow-up work elucidating a unique LTD mechanism in hippocampal electrophysiological recordings ostensibly driven by upstream increases in stress signaling molecules (i.e., adrenaline/noradrenaline) that are known to be amplified in the dependent state. Other work from Paulo et al. utilized a high affinity ligand for nicotinic α7 receptors (α-bgtx-conjugated beads) to identify 55 interacting proteins that were not present in α7-KO negative control samples [41]. The majority of the α7 receptor interactome were annotated to two pathways: (1) cell structure/protein trafficking and (2) signal transduction. Of interest, they identified multiple proteins related to GPCR signaling and phosphorylation, suggesting a more complex role for the α7 receptor in nicotine dependence beyond its canonical function as a calcium channel. McClure-Begley et al. performed similar analyses of the nicotinic α4β2 receptor using a β2 subunit antibody for immunopurification in conjunction with receptor subunit knockout mice [42] and identified 208 proteins in the α4β2 interactome. Subsequent use of this approach in post-mortem cortical tissue from nicotine-dependent mice and human subjects revealed 17 dose-dependent nicotine-induced changes in protein interactions in mice, with eight of these, including CaMKIIα, recapitulated in tissue from human smokers. The molecular relevance of the proposed interaction was recently elucidated by the Picciotto group using both *in vitro* and *in vivo* preparations [43]. These studies provide an excellent example of the utility of interactome studies in identifying novel protein interactions that may serve as the basis for developing precision medicine. Ongoing work in our laboratory is exploring the means by which immunoprecipitation methods may be applied towards the study of protein-protein interactors in mechanisms that mediate post-translational modifications (e.g., protein kinases) [44].

## 5. Phosphoproteomics: Signaling-Driven Phosphorylation States Underlying Addictive Behaviors

The detection of phosphoproteins has provided addiction scientists with a useful tool for measuring changes in activated states that may be devoid of changes in respective protein levels. Protein phosphorylation constitutes one of the most common post-translational modifications in protein biology, whereby the enzymatic addition or subtraction of a phosphate group onto nucleophilic residues (serine, threonine, or tyrosine) can alter the structural conformation of a protein, rendering it active, inactive or otherwise modifying its function. Though not exclusively linked to conformational changes, protein phosphorylation has been shown to serve as a molecular switch influencing a wide range of biological activity including signal transduction, cell differentiation/proliferation, protein-protein/-gene interactions and subcellular localization. Indeed, a large number of hypotheses recognize the importance of protein phosphorylation in directing the flow of molecular signaling, converging on key regulators of gene transcription (e.g., the cAMP response element-binding protein, delta fosB), membrane receptors (e.g., GluA1) and other important binding partners (e.g., transmembrane AMPA receptor regulatory proteins) that modulate neuroplasticity [45,46,47]. In this sense, several hundred kinases and phosphatases are encoded in the human genome and display a plethora of substrate targets [48]. A substantial component of receptor-mediated neuronal signaling involves the modulation of kinases and phosphatases, and in this regard, broad-scale approaches to the phosphoproteome are poised to contribute unique information into the role of phosphorylation states in addiction pathology.

Several aspects of the phosphoproteome are conducive to the identification of novel addiction-related targets. First, global assessments of the phosphoproteome often reveal an abundance of phosphoproteins involved in the regulation of phosphorylation states [49]. Indeed, numerous studies have elucidated the strong therapeutic potential of kinase inhibitors in oncology by targeting the phosphorylation-related constructs that drive cancer malignancy and metastasis [50]. Structurally, the conserved catalytic domain can be targeted with small-molecule inhibitors that exploit the biochemical features of the kinase core, leading to the generation of both reversible and irreversible inhibitors [50,51]. Likewise, protein phosphatases provide a complementary approach for addressing dysregulated phosphoproteins, and a number of successful drugs have been designed as substrate mimetics to target the active site of tyrosine phosphatase. Finally, a phosphoproteomics approach identifies unique peptide sequences in downstream protein substrates that are targeted by these mechanisms. The combined analysis of phospho-enriched proteins together with the unmodified proteome extrapolate well with identified nodes and canonical pathways, increasing the confidence of the candidate target [52,53]. Classical or allosteric approaches to modify the function of proteins by manipulating their phosphorylation state also provides an alternative approach to therapeutic development. In this sense, phosphoproteomics provides multiple avenues to gain insight into drug-related mechanisms of neuroplasticity and the development of precision medicine [54].

Presently, there are only a few studies that have applied a discovery-based phosphoproteomics approach in addiction models. Our laboratory recently utilized metabolic labeling procedures to quantify phosphopeptides enriched from the prefrontal cortex of rats receiving the psychedelic compound phencyclidine (PCP) [49]. We applied the analysis across experimental groups, allowing for the comparison of drug-induced effects relative to those incurred by an additional assessment of sensorimotor gating (i.e., prepulse inhibition) for evaluating schizophrenic-like phenotypes. In total, we identified approximately 120,000 phosphopeptides across experimental groups, 99,810 of which were confidently quantified using a nitrogen-heavy feeding protocol as an internal standard [55]. Overall, PCP treatment resulted in the downregulation of phosphorylation events that were enriched for LTP signaling. While consistent with the drug’s mechanism of action (i.e., NMDA receptor blockade), the comparison of individual phosphosites revealed increased phosphorylation of proteins that regulate glutamatergic tone. Notably, the Serine 26 phosphosite on the light chain of the cysteine/glutamate transporter (SLC7A11) was hyperphosphorylated in PCP-treated rats undergoing prepulse inhibition testing, and site mutagenic procedures confirmed the role of this phosphosite in reducing glutamatergic uptake. This suggests that multiple levels of analysis (pathway, phosphosites and site mutagenics) are necessary to provide optimal insight into the molecular mechanisms influencing drug-induced glutamatergic dysregulation.

Another exceptional study by Rich et al. [56] utilized a label-free discovery approach to compare the amygdalar phosphoproteome in cocaine self-administering rats experiencing either drug-cue extinction or reconsolidation procedures. Microwave irradiation and phospho-enrichment procedures led to the quantification of phosphopeptides from 355 unique proteins, of which approximately 80 were compared across treatment conditions using SRM analysis. Interestingly, the authors reported 5 phosphopeptides that were regulated in the opposite manner by extinction and reconsolidation. This presents an attractive pattern of activation given that memories enter a labile state in which protein synthesis is required to re-stabilize drug-cue associations into long-term memory [57]. As extinction itself is driven by independent learning processes [58], molecules displaying less overlap between these conditions were hypothesized to serve as better therapeutic targets for ameliorating the effects of drug-cue memories. In this regard, a novel phosphosite (Serine 331) in the LTP-associated molecule CaMKIIα was examined further with site mutagenic procedures, ultimately showing that activation of this site reduced CaMKII catalytic activity. The localized infusion of CaMKII inhibitors into the amygdala prevented the reinstatement of cocaine-seeking, providing support for the assertion that CaMKIIα is critically involved in the retention of drug-cue memories that can trigger relapse. These findings are also in agreement with our recent work, displaying a similar pattern of upregulated CaMKIIα activity, albeit with the autophosphorylation site Threonine 286 in the dorsomedial prefrontal cortex of alcohol-dependent rats that may influence cognitive deficits during withdrawal [59]. Indeed, recent studies have demonstrated that genetic abrogation of Threonine 286 dysregulates the phosphorylation of other sites on CaMKII and further alters synaptic protein-protein interactions associated with neurodevelopmental disorders [60]. Pharmacological approaches may then target these regulatory phosphosites with peptidomimetic approaches that are shown to uniquely interface phosphoproteins [61].

## 6. Neuropeptides: Peptide Signals Driving Neuroplasticity in Addiction

Neuropeptides play a central role in the development and persistence of addictive behaviors [62]. For example, exogenous opiates such as oxycodone and heroin directly act on neuropeptide receptors in the brain to produce robust dopamine release, which is characteristic of drugs of abuse. Thus, both antagonists (naloxone) and partial agonists (buprenorphine) of these opiate receptors remain some of the few effective FDA-approved therapeutics for clinical use in treating drug addiction [63]. Research efforts studying the role of neuropeptides in addiction have not been limited to the opioid class, and a critical role has been established for a number of distinct neuropeptides, including (but not limited to) oxytocin [64], neuropeptide Y [65], substance P [66], and corticotropin releasing factor [67]. Notably, cocaine and amphetamine-regulated transcript (CART) represents a neuropeptide that was discovered due to its substantially enhanced expression following exposure to specific drugs of abuse [68].

Neuropeptides are short sequences of amino acids that can act like neurotransmitters, but with some critical distinctions that underlie their importance as potential druggable targets. While both neurotransmitters and neuropeptides are packaged in vesicles and released during neuronal activity, neuropeptides are expressed discretely throughout the CNS to facilitate specific behavioral responses. Neurons and glia have robust, rapid reuptake systems for most neurotransmitters, allowing the recycling of these molecular signals to reduce energy consumption in the CNS. In contrast, substantially more time and energy is required to produce a neuropeptide [69]; first, the pro-neuropeptide gene must be transcribed and translated into a pro-peptide sequence, then it is processed into active neuropeptides at consensus KK/KR sites by specific serine hydrolases. While neuropeptides are typically packaged into large dense-core vesicles that exhibit a longer latency for release and require prolonged stimulation [70], the mechanisms that regulate the vesicular loading and activity-dependent release of specific neuropeptides remain under investigation. Neuropeptides almost universally act at G-protein coupled receptors that do not directly produce action potentials [71], but instead modify neuronal responsivity by altering second messenger signaling (e.g., cAMP, IP3), phosphorylation states (via kinases, phosphatases), and protein levels (through changes in transcription, translation). In many cases, these factors cause neuropeptides to operate on longer time scales than typical neurotransmitters and allow them to exert hormone-like effects in the CNS that facilitate long-term behavioral changes.

Most established neuropeptides currently implicated in addiction were discovered prior to the development of modern proteomic approaches [72]. The idea that hormones acting as chemical messengers could transmit information over long distances originated over a hundred years ago, and the chemical identities of neuropeptide hormones implicated in addiction were discovered in the last century: oxytocin (1953), substance P (1971), enkephalins (1975), dynorphin (1979), and corticotrophin releasing factor (1981) [73,74,75,76,77]. In each case, the purification of each neuropeptide was performed from bulk tissue homogenates using sequential fractionation approaches, with a subsequent evaluation of each fraction for activity in basic *ex vivo* bioassays including the induction of uterine contractions, intestinal contractions, and secretion of other hormones. Likewise, their localization within the CNS was evaluated using antibody-based immunohistochemical techniques. The technical limitations of these approaches have biased studies toward high abundance neuropeptides, and as a result low abundance signals potentially dysregulated by abused drugs and other psychiatric disorders remain understudied.

Modern genetic and proteomic technologies have facilitated more comprehensive studies of neuropeptides. The human genome project (and corollary projects in related species) has identified over 70 genes that contain sequences capable of producing more than 1000 potential neuropeptides [78]. After considering the multitude of potential post-translational modifications, the technical challenges facing neuropeptidomics research becomes evident. The identification of these theoretical neuropeptides using matrix assisted laser desorption ionization (MALDI) or high performance liquid chromatography/electrospray ionization (HPLC/ESI) mass spectrometry has proven to be challenging, with many outstanding reviews outlining the technical issues with neuropeptide mass spectrometry [79,80,81,82,83]. Here, we highlight a few studies that have utilized a peptidomic approach to address critical biological questions regarding the role of neuropeptides in addiction and other psychiatric disorders.

### 6.1. Where Are Neuropeptides Located in the CNS?

Neuropeptide expression in discrete regions allows them to exert their effects on specific circuits to produce distinct behavioral outcomes. In the striatum, dopaminergic neurons regulate excitatory and inhibitory signaling to facilitate drug reward. Neuropeptides produce maladaptive effects by acting on specific neurons in distinct locations in the striatum and other limbic regions, ultimately contributing to drug craving and compulsive behaviors that influence relapse. Thus, the spatial location of precise neuropeptides plays a critical role in the development of addiction.

Hishimoto et al. investigated the localization of neuropeptides in the striatum using MALDI imaging mass spectrometry (MALDI-IMS) [84]. Mice exposed to acute nicotine were evaluated for changes in two distinct circuits in the striatum: (1) projections to the substantia nigra pars compacta (direct pathway) containing substance P and dynorphin, and (2) projections to the substantia nigra pars reticulate (indirect pathway) containing enkephalins. Using traditional immunohistochemical staining techniques, it is possible to distinguish (but not physically separate) these two pathways *in situ*. Hishimoto used MALDI-IMS with sufficient resolution (200 μm) to identify these structural features using substance P and enkephalin as molecular markers, and thereby demonstrated that nicotine administration decreased nigral substance P levels while increasing enkephalin levels. In total, 768 features were identified from mouse striatal spectra, and more than half of these were significantly regulated by nicotine. More importantly, they found that nicotine produces a negative correlation between substance P and other *m*/*z* species identified in those samples, whereas a positive correlation emerged between *m*/*z* identified in regions with enkephalin. These results suggest a more broad-scale remodeling of the direct (substance P) and indirect (enkephalin) pathways in the striatum, working in concert to facilitate behavioral changes produced by nicotine exposure.

Recent work has also identified the habenula as an important regulator of nicotine dependence. The habenular nuclei are morphologically and biochemically distinct structures that innervate reward circuitry: the medial habenula is thought to play a critical role in the maintenance of nicotine consumption, while the lateral habenula drives the aversive effects of the drug [85]. Immunohistochemical studies suggest that these regions have distinct repertoires of densely expressed neuropeptide receptors. For these reasons, Yang et al. utilized a tissue-stabilized liquid chromatography tandem mass spectrometry (LC-MS/MS) approach to identify the complimentary neuropeptidome in the habenula [86]. They identified 331 potential neuropeptides in this region, including many that were exclusive to either the medial (136 peptides) or lateral (51 peptides) habenula. While some of these peptides have well established roles in reward circuitry, the functions of others have not been widely investigated, suggesting that further studies investigating their involvement in addiction are warranted. Some of the neuropeptides identified remain more nebulous, as their putative receptors have not been fully established (e.g., secretogranins). Collectively, these results suggest that a comprehensive analysis of neuropeptide changes in emerging addiction structures such as the habenula will uncover new druggable targets for altering addictive behaviors.

### 6.2. Which Neuropeptides Get Released for Extracellular Signaling in the CNS?

In addition to exhibiting differences between brain structures, neuropeptide levels vary dramatically within brain structures at the subcellular level. Genetic sequence analyses predict that over 1000 potential neuropeptides may exist, with many having already been identified in CNS tissue using peptidomic approaches. In the striatum, Ye et al. identified 419 *m*/*z* that correspond to potential neuropeptides and discovered that many of these peptides were regulated by behavioral manipulation [87]. Specifically, they found that proSAAS derived peptides (big LEN, PEN, and little SAAS) were decreased in mice that were unfed (hungry). The microinjection of big LEN dramatically decreased food intake in regularly fed mice, demonstrating a clear functional link between big LEN and satiety, and suggesting that big LEN acts in the striatum through an extracellular, receptor-mediated mechanism.

For most studies, the analysis of neuropeptide content has typically been performed on samples from bulk tissue. As illustrated by analogous work studying neurotransmitters and endocannabinoid lipids in the CNS, there are some potential limitations to using this approach [88,89]. Briefly, it is not easy to distinguish neuropeptides found in vesicles and extracellular spaces (active signals) from those found in the endoplasmic reticulum and lysosomes (deactivated). Even with advanced sample handling technology [90,91], post-mortem peptide degradation remains a significant concern. In addition to these technical issues, environmental factors such as stress and circadian rhythm have a substantial impact on peptide hormonal levels. Given the large number of potential neuropeptides identified in comprehensive neuropeptidomics approaches, there is an unmet need for techniques that refine the population of peptides for identifying high-value targets.

**Figure 2:**
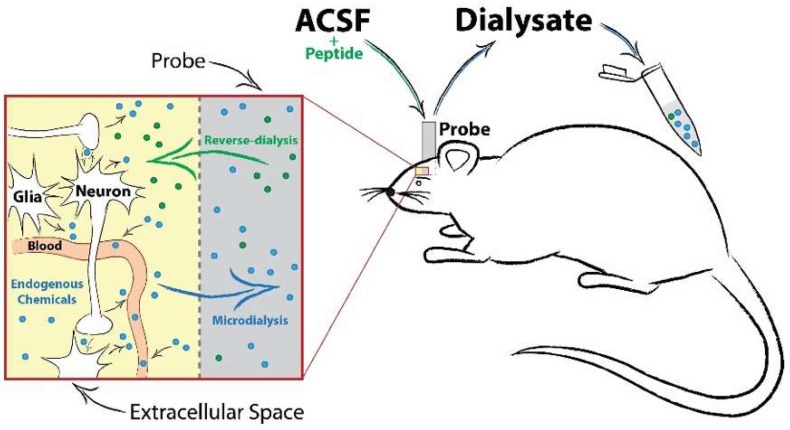
Schematic of an *in vivo* microdialysis probe setup, neurochemical diffusion and sample collection. Microdialysis sampling involves the implantation of a small-diameter probe into the brain region of interest. Artificial cerebrospinal fluid (ACSF) is perfused continuously into the probe, creating a concentration gradient at the semi-permeable membrane tip. This allows for the passive diffusion of extracellular transmitters (conventional dialysis) or solubilized compounds or drugs (reverse-dialysis) in the ACSF to freely enter or exit the probe. A timeline of collection can then be implemented to explore changes in neurotransmission before and after an experimental manipulation.

Sampling using *in vivo* microdialysis addresses many of these concerns and provides a useful approach for identifying signaling-competent peptides in the CNS during drug exposure [88,92] (Figure 2). Through the implantation of a semi-permeable probe located within a specific brain structure, microdialysis allows for repeated sampling from awake and behaving animals. Moreover, microdialysate samples contain molecules in equilibrium with the extracellular space, providing an index for signaling-competent molecules available for binding to cell surface receptors. Due to the nature of this sampling approach, mass spectrometry analyses of microdialysates typically contain a lower neuropeptide diversity and content than traditional bulk tissue analyses. However, the neuropeptides identified using this technique have a greater likelihood of producing receptor-mediated effects, and they can be readily assessed for bioactivity using this approach. For example, Haskins et al. performed a peptidomic analysis of microdialysate samples collected from the striatum [93]. In this study, they used an untargeted LC-MS/MS approach and identified 3349 *m*/*z* features released in the rat striatum. From these data, they identified 29 potential neuropeptides from 6 different genes, including two peptides from enkephalin (PENK 198–207 and BAM 8–22). A similar study by Bernay et al. used multiple microdialysis and mass spectrometry approaches to more comprehensively investigate the striatal neuropeptidome and identified 97 peptides from these samples, including additional pro-enkephalin peptides PENK 114–133 and PENK 239–260 [94]. Given their lack of homology with met-enkephalin, these PENK peptides are unlikely to activate traditional opiate receptors and likely would exert their bioactivity through an alternative mechanism. Taken together, these studies indicate that pro-enkephalin biology may extend beyond traditional opioid receptor signaling, thereby warranting further investigation.

The reverse-dialysis of neuropeptides, a complementary technique to microdialysis, provides a rapid and effective readout for establishing *in vivo* bioactivity. Whereas microdialysis facilitates the diffusion of peptides in the brain to dialysate fluid in the probe, reverse-dialysis takes advantage of the ability to introduce potentially active substances into the dialysate fluid (e.g., neuropeptides) for subsequent distribution in the brain to their biological targets [89]. Thus, reverse-dialysis allows for the site-specific application of a potential neuropeptide to produce local effects on the CNS that can be assessed by changes in release of traditional neurotransmitters (e.g., glutamate, GABA, dopamine) implicated in addiction circuitry. Both Haskins et al. and Bernay et al. effectively used this approach to evaluate the bioactivity of their respective PENK peptides [93,94], demonstrating that both PENK 114–133 and PENK 198–207 increased glutamate release while suppressing GABA. Alternatively, PENK 239–260 produces robust increases in glutamate with no significant effect on GABA, whereas BAM8–22 activates GABA release. Collectively, these results suggest greater complexity in pro-enkephalin signaling than has been appreciated previously and implicate alternative strategies for targeting reward circuitry. Given the importance of the opioid system as a treatment for drug addiction, these pro-enkephalin peptides remain an important unanswered area in the study of addiction. More broadly, many of the neuropeptide genes implicated in addiction contain multiple peptides that can be identified *in vivo* using peptidomic strategies, and these peptides should be explored further as potential druggable targets.

### 6.3. How do We Elucidate Neuropeptide Bioactivity?

The transition from peptide identification to target discovery remains a critical bottleneck in neuropeptidomics. As illustrated in the studies above, traditional behavioral and molecular biology approaches can establish a functional role for neuropeptides identified using mass spectrometry. As reported by Ye et al., peptides can be site-specifically infused into brain structures to evaluate behavioral outcomes and establish a functional link with the underlying biology [87]. Likewise, peptides can be site-specifically administered by reverse-dialysis to investigate changes in levels of traditional neurotransmitters such as glutamate and GABA [93,94]. Both approaches provide invaluable information about the bioactivity of a specific neuropeptide, yet fall short of elucidating specific receptor-mediated mechanisms. The following studies provide examples of different approaches for identifying specific receptors for orphan neuropeptides.

Given the importance of big LEN in food intake [95], Gomes et al. sought to identify the receptor responsible for its behavioral effects. Using the criteria of targeted receptor subclass (GPCR, G_i/o_) and gene expression information (enriched in hypothalamus and Neuro2A cells), they limited the possibilities to four likely targets, ultimately revealing GPR171 as the endogenous big LEN receptor. Cells overexpressing GPR171 showed characteristic G_i/o_-mediated decreases in cAMP levels following exposure to big LEN, and GPR171 siRNA delivered to the hypothalamus led to increased food intake in mice. Subsequent studies have identified a role for big LEN and GPR171 in anxiety-like behavior and fear conditioning [96], suggesting this system may influence addiction pathology. A small molecule agonist of GPR171 is now available [97], and future studies will evaluate its viability as a therapeutic target.

The proenkephalin-derived peptide BAM 8–22 has previously been identified as an endogenous anti-nociceptive peptide [98], and thus may counteract the actions of other opioid peptides. To identify receptor(s) that mediate the effects of BAM 8–22 and other orphan peptides, Kroeze et al. codon-optimized the Tango assay of Barnea et al. [99] and developed a method for the simultaneous and parallel interrogation of the entire human nonolfactory GPCRome (parallel receptorome expression and screening via transcriptional output, with transcriptional activation following arrestin translocation; PRESTO-Tango). Although GPCRs can signal through a multitude of intracellular second messenger systems, thw sustained activation of nearly all GPCRs leads to the binding of β-arrestin and internalization, thereby providing a universal assay platform for screening a variety of receptors concurrently. Each Tango construct is engineered to promote β-arrestin recruitment and ultimately drive the expression of luciferase upon the binding of the ligand. Kroeze et al. designed and validated Tango constructs for nearly the entire human GPCRome (83%), enabling them to screen a single peptide against a wide range of potential receptors, including over 100 orphan GPCRs. Using this technology, BAM 8–22 was identified as a potent activator of Mas-related G-protein coupled receptor member X1 (MRGPRX1) and other members of this receptor family. This work helped facilitate the development of potential analgesics targeting MRGPRX1 [100], which may have reduced abuse liabilities as they do not activate the mu-opioid receptor. Further exploration of the MRGPRX family suggests that many opioid scaffolds can potently activate MRGPRX receptors [101], and that these receptors may be responsible for some of the undesirable side effects such as opioid tolerance [102]. More broadly, this technology provides a unique approach for uncovering links between peptidomics and orphan receptors and establishes a foundation for the hit-to-lead optimization of novel druggable targets for addiction and other psychiatric disorders.

## 7. Conclusions and Future Directions

The population of drug-addicted individuals continues to expand in the United States and worldwide, but the dearth of available treatment options has impeded progress in addressing this escalating burden. As our understanding of the neurobiology of addiction has evolved, it has become increasingly apparent that subtle changes in discrete brain circuits facilitate maladaptive behaviors associated with mood and cognition that ultimately lead to relapse and sustained drug use. Thus, the continued development of novel mass spectrometric-based methods such as cell-specific labeling [103] and single cell proteomics [104] in combination with currently available neuroscientific tools is critical for the detection of molecular drivers of addiction and their subsequent translation into viable clinical treatments. Future studies utilizing these approaches will undoubtedly improve our ability to interrogate brain circuitry, ultimately forging a unique path for identifying novel druggable targets of addiction and related psychiatric disorders.
